# 

**Published:** 1894-11

**Authors:** 


### CONTENTS.

ORIGINAL ARTICLES—

The Hygienic and Dietetic Management of Phthisis. By L. P. Barbour, M. D., Thomasville, Ga.......... 581

Transfusion of Blood and Infusion of Saline Solutions: Indications, Methods and Results. By Valentine H. Taliaferro, M. D., Atlanta, Ga.......... 589

Cataract—Clinical Lecture of Frank Trester Smith, A. M., M. D., Professor of Diseases of the Eye in the Chattanooga Medical College. Reported by E. B. Clark.......... 608

SOCIETY REPORTS—

Proceedings of the College of Physicians, of Philadelphia. Case of Gunshot Wound of Abdomen and Lung. By Lewis W. Steinbach, M. D.......... 610

Case of Gunshot Wound of Liver and Lung. By Thomas S. K. Morton, M. D.......... 612

BOOK REVIEWS—

Landmarks in Gynecology. By Byron Robinson, B. A., M. D., Chicago, Ill.......... 617

Hand-book of Medical Microscopy for Students and General Practitioners. By James E. Reeves, M. D.......... 617

### CONTENTS.—Continued.

SELECTIONS AND ABSTRACTS—

The Treatment of Ear-Disease by the General Practitioner. By B. Alex. Randall, M. D., Philadelphia........................ 618

A Case of Neuritis Simulating Progressive Muscular Atrophy Associated with Nystagmus and Increased Knee Jerks. By Harold N. Moyer, M. D., Chicago, Ill.................................. 621

How to Find Out if a Case of Gonorrhœa is Actually Cured....... 624

Alcohol in Neurasthenia. By Græme M. Hammond, M. D., New York.................................................. 624

New Methods of Treating Hydrocele.............................. 626

Femoral Epiplocele in a Woman—Radical Operation. By William J. Taylor, M. D.............................................. 627

EDITORIAL—

Bias of Medical Testimony Regulated by a Commission of Experts, 629

EDITORIAL NOTES..........................................631—633

PRESCRIPTION DEPARTMENT—

Tonsillitis—Dipsomania—Diuretic and Rheumatic—For Pyelitis.. 634

